# Synthesis and Oxygen Storage Capacities of Yttrium-Doped CeO_2_ with a Cubic Fluorite Structure

**DOI:** 10.3390/ma15248971

**Published:** 2022-12-15

**Authors:** Yaohui Xu, Liangjuan Gao, Zhao Ding

**Affiliations:** 1 Laboratory for Functional Materials, School of New Energy Materials and Chemistry, Leshan Normal University, Leshan 614004, China; 2 National Engineering Research Center for Magnesium Alloys, College of Materials Science and Engineering, Chongqing University, Chongqing 400044, China; 3 College of Materials Science and Engineering, Sichuan University, Chengdu 610065, China; 4 Leshan West Silicon Materials Photovoltaic and New Energy Industry Technology Research Institute, Leshan 614000, China

**Keywords:** CeO_2_, rare-earth, doping, hydrothermal, impregnation, oxygen storage capability

## Abstract

Doping CeO_2_ with Y cations was achieved in this study using three strategies: doping only during the hydrothermal process (H-Y-doped CeO_2_), doping only during the impregnation process (I-Y-doped CeO_2_), and doping during both the hydrothermal and impregnation processes (H/I-Y-doped CeO_2_). During the three synthesis strategies of Y-doped CeO_2_, these Y ions could be incorporated into the CeO_2_ lattice in the +3 state while holding the cubic fluorite structure, and no impurity phases were detected. Pure CeO_2_ crystal itself contained a certain number of intrinsic *V*_O_ defects, and Y-doping was beneficial for the creation of extrinsic *V*_O_ defects. The relative concentrations of *V*_O_ defects were quantified by the values of *A*_592_/*A*_464_ obtained from Raman spectra, which were 1.47, 0.93, and 1.16 for the H-Y-, I-Y-, and H/I-Y-doped CeO_2_, respectively, and were higher than that of the undoped one (0.67). Moreover, the OSCs of the three Y-doped CeO_2_ were enhanced, and the sequence of OSCs was: H-Y-doped CeO_2_ (0.372 mmol/g) > H/I-Y-doped CeO_2_ (0.353 mmol/g) > I-Y-doped CeO_2_ (0.248 mmol/g) > Undoped CeO_2_ (0.153 mmol/g); this result was in good agreement with the Raman spectroscopy results.

## 1. Introduction

With the continuous development of science and technology, the development and utilization of energy and resources have become a hot topic [1,2,3]. Rare-earth elements, known as “industrial monosodium glutamate”, “industrial vitamin”, and “mother of new materials”, are precious strategic metal resources. Cerium (Ce) is the most abundant rare-earth element in the Earth’s crust. Its common valence states are +3 and +4, and the corresponding oxides are cerium sesquioxide (Ce_2_O_3_) and cerium dioxide (CeO_2_) [4,5,6]. Ce_2_O_3_ is unstable in air and easily oxidizes to CeO_2_. Interestingly, there are not only Ce^4+^ ions in CeO_2_ crystals but also trace amounts of Ce^3+^ ions. The oxidation/reduction cycle composed of Ce^3+^ and Ce^4+^ states (Ce^3+^↔Ce^4+^) enables CeO_2_ to store and release oxygen, referred to as oxygen storage capability (OSC). In other words, CeO_2_ can release oxygen under reducing conditions, forming nonstoichiometric oxides CeO_2−*x*_, and the CeO_2−*x*_ can store oxygen by filling oxygen vacancies under oxidizing conditions [7,8]. In the atmosphere or an oxygen-rich environment, CeO_2_ can store some oxygen in its own lattice, and these stored oxygen atoms can be released quickly when the partial pressure of ambient oxygen decreases. Precisely because of this ability, CeO_2_ is considered an excellent catalyst in CO_2_ methanation [9,10], hydrodeoxygenation of xylitol and fatty acids [11,12], NO*_x_* conversion [13,14], and so on.

Doping the CeO_2_ lattice with other metallic elements has been proven to be an effective method to improve the OSC of CeO_2_. These include rare-earth elements (Sm [15], La, Pr, Nd, and Pm [16]), transition-metal elements (Fe [17], Ni [18], Co [19], Mn, and Cu [20]), earth-alkaline elements (Be, Mg, Ba, and Sr [21]), and noble-metal elements [22]. Among many impurity elements, the incorporation of rare-earth elements into the CeO_2_ lattice should be relatively easy because the impurity rare-earth metallic ions and Ce ions have a similar ionic size and electronic structure. To date, numerous methods have been used to synthesize rare-earth-doped CeO_2_, including the co-precipitation method [23], metalorganic chemical vapor deposition (MOCVD) [24], the solid-state reaction (SSR) method [25], the sol–gel method [26], and flame spray pyrolysis [27]. Among the many methods for doping CeO_2_, the hydrothermal method is considered to be one of the most effective. A Teflon-lined stainless-steel autoclave is employed in the hydrothermal synthesis process, and the closed reaction environment can breed a high-pressure environment under the action of heating, which is conducive to integrating impurity ions into the lattice of CeO_2_ or cerium precursor [28,29]. In addition, the impregnation method also has attracted an increasing amount of attention recently because the operation of this technique is simple and efficient, especially for doping onto the CeO_2_ surface [30,31].

In this work, the Y element was introduced into the CeO_2_ lattice by three routes in the same system, using the same cerium source (Ce(NO_3_)_3_∙6H_2_O) and dopant (Y(NO_3_)_3_∙6H_2_O). The first route involved the incorporation of Y ions during only the hydrothermal process, whereas the second route involved the incorporation of Y ions during only the impregnation process, and the third route involved the incorporation of Y ions during both the hydrothermal and impregnation processes. The characterizations of the phase composition, lattice parameters, quantitative concentration of Ce^3+^, and oxygen vacancy of these as-synthesized Y-doped CeO_2_ were investigated and discussed in detail. Moreover, a comparative analysis was also performed. Subsequently, the OSC of CeO_2_ was examined and quantified using O_2_ temperature-programmed desorption (O_2_-TPD) measurements.

## 2. Experimental Section

### 2.1. Starting Materials

Ce(NO_3_)_3_∙6H_2_O (99.95%) and Y(NO_3_)_3_∙6H_2_O (99.99%) were supplied by Aladdin Co., Ltd. (Shanghai, China) and Maclin Biochemical Technology Co., Ltd. (Shanghai, China), respectively. Ethylene glycol (EG, 99.5%) and acid orange 7 (AO7, 97.0%) were supplied by Chengdu Kelong Chemical Co., Ltd. (Chengdu, China) and Tokyo Chemical Industry Co., Ltd. (Tokyo, Japan), respectively. Distilled water was used in all experiments. All major chemicals were used as received without further purification.

### 2.2. Synthesis of Y-Doped CeO_2_ Powders

**Figure 1:**
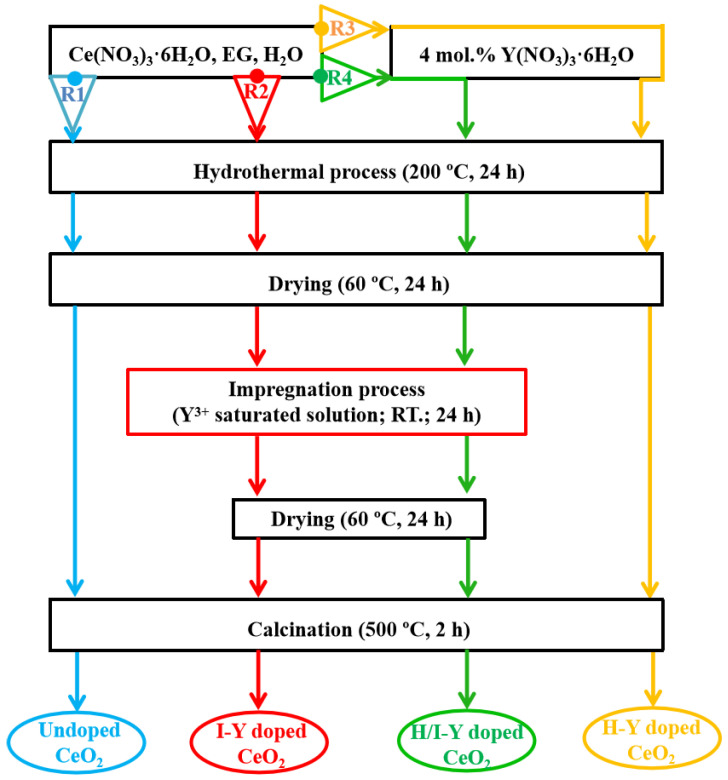
Schematic illustration of the synthesis of undoped and Y-doped CeO_2_ using three routes.

A flow chart of the synthesis procedures employed for the undoped and Y-doped CeO_2_ samples is shown in Figure 1. The reference CeO_2_ without Pr-doping was prepared by the hydrothermal process using Route 1 (R1), as shown in Figure 1, denoted as Undoped CeO_2_. The CeO_2_ samples doped with Pr cations during only the impregnation process or the hydrothermal process were synthesized as shown in Route 2 (R2) and Route 3 (R3) of Figure 1, denoted as I-Y-doped CeO_2_ and H-Y-doped CeO_2_, respectively, while the one doped during both the impregnation and hydrothermal processes was synthesized as shown in Route 4 (R4) of Figure 1, denoted as H/I-Y-doped CeO_2_.

Undoped CeO_2_ was synthesized using R1: 4.0 mmol Ce(NO_3_)_3_∙6H_2_O, 25 mL EG, and 5 mL distilled water were added into a 50 mL Teflon-lined stainless-steel autoclave and sealed at 200 °C for 24 h. Afterward, the Ce precursor was collected, washed, and dried in turn. Finally, Undoped CeO_2_ was obtained by subsequent calcination in air at 500 °C for 2 h.

I-Y-doped CeO_2_ was synthesized using R2: the Ce precursor synthesized in R1 was impregnated into a saturated solution of Y^3+^ ions at room temperature for 24 h. After filtration and drying, the I-Y-doped CeO_2_ was obtained by subsequent calcination in air at 500 °C for 2 h.

H-Y-doped CeO_2_ was synthesized using R3: 3.84 mmol Ce(NO_3_)_3_∙6H_2_O, 0.16 mmol Y(NO_3_)_3_∙6H_2_O, 25 mL EG, and 5 mL distilled water were added into a 50 mL Teflon-lined stainless-steel autoclave and sealed at 200 °C for 24 h. Afterward, the Ce/Y precursor was collected, washed, and dried in turn. Finally, H-Y CeO_2_ was obtained by subsequent calcination in air at 500 °C for 2 h.

H/I-Y-doped CeO_2_ was synthesized using R4: the Ce/Y precursor synthesized in R3 was impregnated into a saturated solution of Y^3+^ ions at room temperature for 24 h. After filtration and drying, the H/I-Y-doped CeO_2_ was obtained by subsequent calcination in air at 500 °C for 2 h.

### 2.3. Characterization

The crystallographic phases of the samples were characterized by X-ray diffraction (XRD, DX-2700). The surface composition and binding energy of the CeO_2_ samples were determined by X-ray photoelectron spectroscopy (XPS, ESCALAB 250Xi, Thermo Scientific, Waltham, MA, USA). The oxygen vacancy (*V*_O_) defects of the CeO_2_ samples were characterized using a Raman spectrometer (LabRAM Aramis, Horiba Jobin-Yvon, Paris, France) with a He–Cd laser of 325 nm, and the exposure time for the measurement set was 60 s.

### 2.4. OSC

The OSC of CeO_2_ was estimated using O_2_ temperature-programmed desorption (O_2_-TPD) measurements, which were carried out in a plug-flow microreactor system (TP5000) with a thermal conductivity detector, and the amount of O_2_ desorption during the process was measured by the thermal conductivity detector. About 0.1 g of CeO_2_ powder was activated using an air stream at 400 °C for 30 min, then moved into He and cooled, then exposed to an air stream for 30 min at 120 °C, followed by purging with a He stream to remove the excess O_2_. Finally, the surface oxygen desorption was conducted at a flow rate of He (10 mL/min) while the temperature was raised to ~900 °C (10 °C/min).

## 3. Results and Discussion

**Figure 2:**
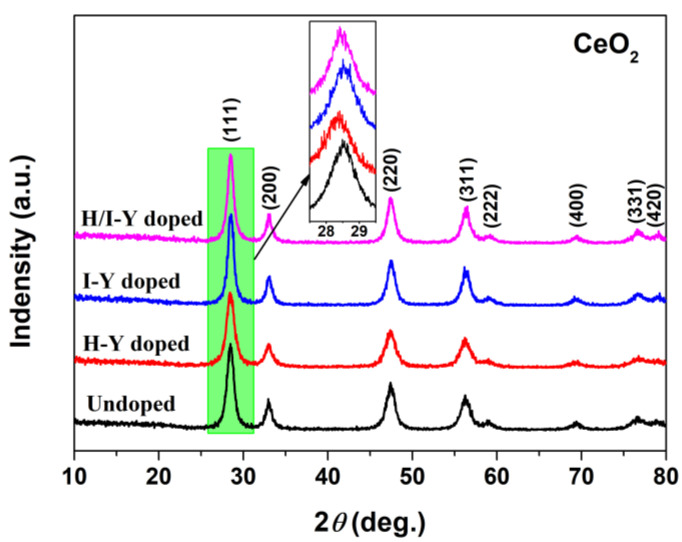
XRD patterns of Undoped and H-Y-, I-Y-, and H/I-Y-doped CeO_2_ powders (the inset shows the (111) reflection shifts using a smaller scan speed of 0.01°/min).

XRD was employed to characterize the phase composition of the as-synthesized undoped and Y-doped samples. Figure 2 shows the XRD patterns of the Undoped CeO_2_ and H-Y-, I-Y-, and H/I-Y-doped CeO_2_ powders. For the XRD pattern of the Undoped CeO_2_, eight well-resolved peaks were observed, which could be indexed to the (111), (200), (220), (311), (222), (400), (331), and (420) planes of cubic CeO_2_ (JCPDS no. 34-0394; fluorite). No additional phases were detected, suggesting pure CeO_2_ had been obtained by the hydrothermal process using route R1 in Figure 1. After the introduction of Y cations in the synthesis process, the XRD patterns of the H-Y-, I-Y-, and H/I-Y-doped CeO_2_ samples exhibited a similar profile to that of the Undoped CeO_2_. However, no peaks for impurity phases such as Y_2_O_3_ were detected, which could be explained as follows: the impurities in Y-doped CeO_2_ samples might exist as highly dispersed or amorphous surface species, or the amount of the Y impurity was low. Another possibility is that the Y cations partially substituted the Ce ions to form a solid solution. The inset in Figure 2 shows that the (111) reflection shifts toward lower 2*θ* values with the incorporation of Y ions, and it can be found that the shift exhibited by the H-Y-doped CeO_2_ sample was the greatest. Moreover, the lattice parameters of CeO_2_ were estimated using Bragg’s equation and summarized in Table 1. It was found that the calculated lattice parameters for H-Y- (5.4242 Å), I-Y- (5.4190 Å), and H/I-Y-doped (5.4227 Å) CeO_2_ were greater than that of the undoped sample (5.4117 Å). These findings implied that the large Y ions (1.02 Å) partially substituted the Ce^4+^ ions (0.97 Å [32]) to form a CeO_2_-based solid solution while holding the cubic fluorite structure of CeO_2_.

**Figure 3:**
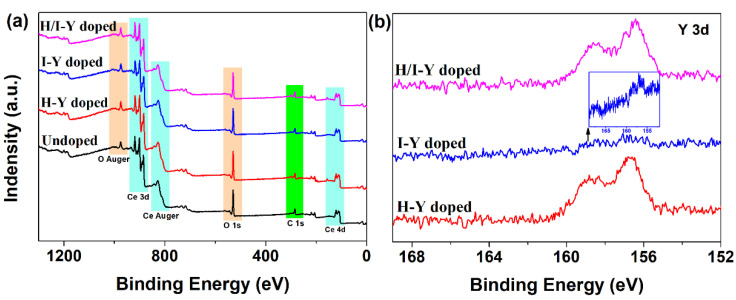
(**a**) Wide-scan XPS spectra of Undoped and H-Y-, I-Y-, and H/I-Y-doped CeO_2_ and (**b**) the corresponding XPS regions of Y 3d for H-Y-, I-Y-, and H/I-Y-doped CeO_2_ (the inset in Figure 3b is an independent drawing of the Y 3d XPS region for I-Y-doped CeO_2_).

In order to probe the possibility of the presence of the Y element in CeO_2_, as well as the chemical state of its presence, XPS was employed to study the Undoped CeO_2_ and Y-doped CeO_2_. Figure 3a shows the wide-scan XPS spectra of the Undoped CeO_2_ and H-Y-, I-Y-, and H/I-Y-doped CeO_2_ powders. As observed in Figure 3a, the XPS profiles of all the samples were similar, dominated by the signals of Ce, O, and C elements, in accordance with a previous report for pure and Y-doped CeO_2_ [33]. However, there was no sign of the Y element at first sight from the wide-scan XPS spectra of the H-Y-, I-Y-, and H/I-Y-doped CeO_2_. To ascertain whether the CeO_2_ contained the Y cations and the Y-doping was real, the corresponding Y 3d XPS regions of the H-Y-, I-Y-, and H/I-Y-doped CeO_2_ were recorded. For the Y 3d XPS regions of the H-Y- and H/I-Y-doped CeO_2,_ we could cleanly identify the Y 3d signal and its unique contour, which is assigned to the trivalent Y ions. However, the Y signal was weak from the Y 3d XPS regions of the I-Y-doped CeO_2_, yet its signal peak of Y 3d could still be identified in graphing alone (inset in Figure 3b).

**Figure 4:**
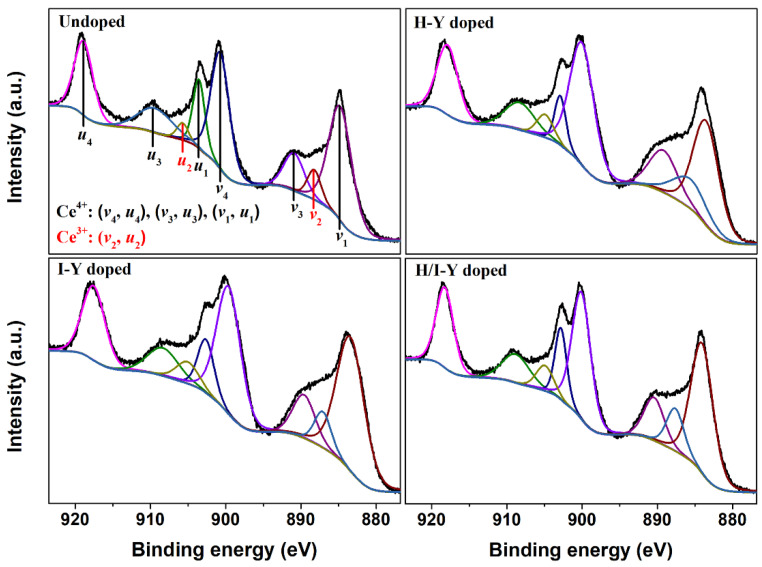
Ce 3d XPS spectra of Undoped and H-Y-, I-Y-, and H/I-Y-doped CeO_2_.

In order to understand the effect of Y-doping on Ce ions in CeO_2_ crystals, the Ce 3d XPS regions of the Undoped CeO_2_ and H-Y-, I-Y-, and H/I-Y-doped CeO_2_ were recorded and fitted, as shown in Figure 4. The Ce 3d core-level XPS of all CeO_2_ samples could be fitted into eight peaks, referring to the 3*d*_5/2_ and 3*d*_3/2_ spin-orbit doublet of Ce cations (including Ce^3+^ and Ce^4+^ ions). The bonds labeled as *v*_2_ and *u*_2_ belong to the spin-doublet term of the Ce^3+^ state, and the bands labeled as *v*_4_, *v*_3_, and *v*_1_ (and those for *u*) are due to the case of the Ce^4+^ state [34]. A quantitative analysis of the concentration of Ce^3+^ ions based on the measured Ce 3d XPS spectra, labeled as [Ce^3+^]_XPS_, could be performed using Equation (1), and the results are summarized in Table 1. The [Ce^3+^]_XPS_ values of the H-Y-, I-Y-, and H/I-Y-doped CeO_2_ were 12.60%, 8.95%, and 11.37%, respectively. These were higher than that of the Undoped CeO_2_ (6.54%), indicating that pure CeO_2_ crystal itself contains a certain number of Ce^3+^ ions and that Y-doping could promote the formation of Ce^3+^ species, especially H-Y-doped CeO_2_, which exhibited the highest [Ce^3+^]_XPS_ values. The Ce^3+^ species in pure CeO_2_ contributed to the OSC of CeO_2_ through the oxidation/reduction cycle composed of Ce^3+^ and Ce^4+^ states (Ce^3+^↔Ce^4+^).

(1) $${\left[Ce^{3+}\right]}_{XPS}(\%)=\frac{A_{u_{2}}+A_{v_{2}}}{A_{u_{4}}+A_{u_{3}}+A_{u_{2}}+A_{u_{1}}+A_{v_{4}}+A_{v_{3}}+A_{v_{2}}+A_{v1}}\times 100$$

where *A*_i_ is the integrated area of the *i*th fitting peak from Ce 3d XPS spectra.

**Figure 5:**
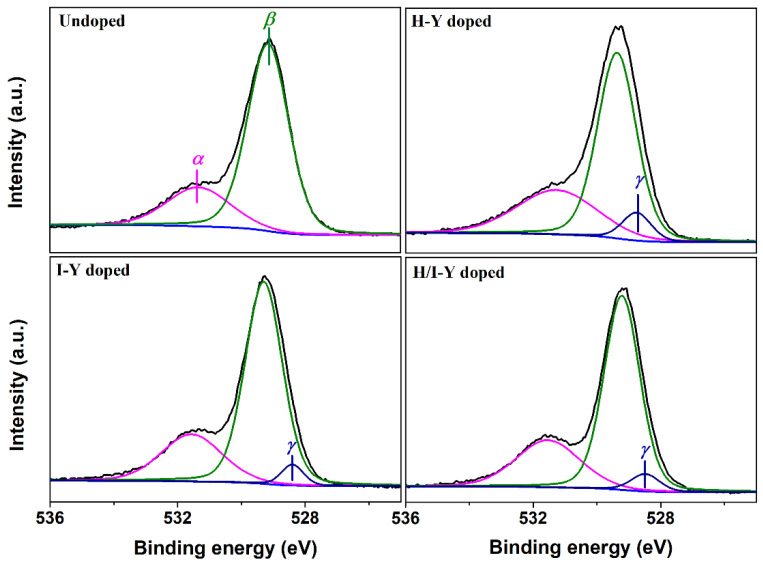
O 1s core-level XPS spectra of Undoped and H-Y-, I-Y-, and H/I-Y-doped CeO_2_.

In order to understand the effect of Y-doping on oxygen ions in CeO_2_ crystals, the O 1s XPS regions of the Undoped CeO_2_ and H-Y-, I-Y-, and H/I-Y-doped CeO_2_ were recorded and fitted, as shown in Figure 5. The O 1s XPS spectrum of the Undoped CeO_2_ was curve-fitted into two peaks: one peak, labeled as *α*, at ~529.2 eV, could be attributable to the lattice oxygen species; the other peak, labeled as *β*, at ~531.4 eV, could be attributable to the chemisorbed oxygen species and/or weakly bonded oxygen species related to the oxygen vacancy (*V*_O_) defects. For the O 1s spectra of the H-Y-, I-Y-, and H/I-Y-doped CeO_2_, a new peak labeled as *γ*, at~528.5 eV, was curve-fitted, which could be assigned to the O-Y species. In addition, the relative oxygen vacancies ratio (labeled as [*V*_O_]_XPS_) could be quantified using Equation (2), and the results are shown in Table 1.

(2) $${\left[V_{O}\right]}_{\mathrm{XPS}}(\%)=\frac{A_{\beta }}{A_{\alpha }+A_{\gamma }+A_{\beta }}\times 100$$

where *A*_i_ is the integrated area of the *i*th fitting peak from O 1s XPS spectra. As observed in Table 1, the [*V*_O_]_XPS_ values of the H-Y-, I-Y-, and H/I-Y-doped CeO_2_ were 30.65%, 26.32%, and 28.72%, respectively, which are higher than that of the undoped sample (24.36%). The results indicate that pure CeO_2_ crystal itself contained a certain number of *V*_O_ species (namely intrinsic *V*_O_ defects) and that Y-doping was beneficial for the creation of *V*_O_ defects, especially H-Y-doped CeO_2_, which exhibited the highest [*V*_O_]_XPS_ values.

**Figure 6:**
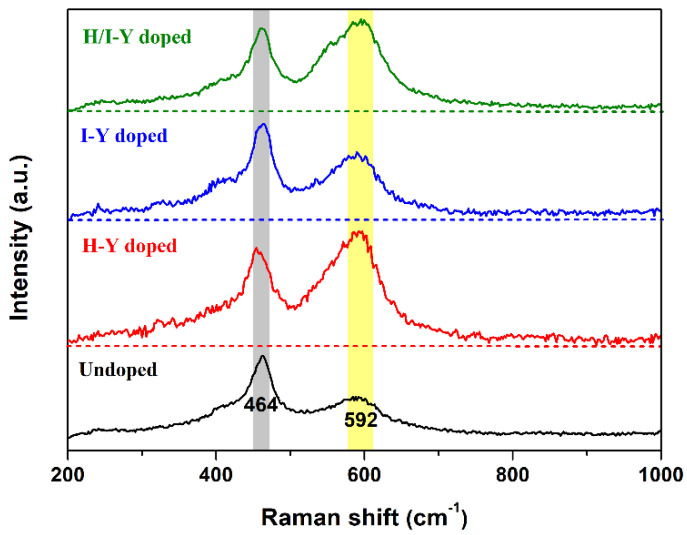
Raman spectra of Undoped and H-Y-, I-Y-, and H/I-Y-doped CeO_2_.

To further investigate the *V*_O_ defects, Raman spectra of CeO_2_ were obtained. Raman spectroscopy is a powerful tool for the structural characterization of metal oxides due to its sensitivity to structural changes, such as *V*_O_ defects. Figure 6 shows the Raman spectra of the Undoped and H-Y-, I-Y-, and H/I-Y-doped CeO_2_. For the Undoped CeO_2_, the spectral envelope in the 200~1000 cm^−1^ range displayed a strong band at 464 cm^−1^ associated with the triply degenerate F_2g_ vibrational mode of CeO_2_ [35,36], while the band located at 592 cm^−1^ was associated with the optical LO mode of substoichiometric CeO_2−*x*_ units, underscoring an increase in *V*_O_ defects [37,38]. Upon Y-doping, increases in the intensity of the bands located at 464 and 592 cm^−1^ were observed for the H-Y-, I-Y-, and H/I-Y-doped CeO_2_, which were associated with the presence of substoichiometric CeO_2-x_, underscoring an increase in *V*_O_ defects. However, the Y^3+^-doping into the CeO_2_ lattice increased its lattice distortion and hence interfered with the vibrations of CeO_2_. Therefore, the Raman band intensities of the Y-doped CeO_2_ were clearly affected by the incorporation of Y^3+^ into the CeO_2_ lattice. Consequently, an alternative approach to estimate the relative concentration of *V*_O_ defects can be adopted by calculating the ratio of the integrated area of the Raman band at 592 cm^−1^ to that of 464 cm^−1^ (labeled [*V*_O_]_Raman_) [39,40]. The values of *A*_592_/*A*_464_, that is, the relative concentration of oxygen vacancies ([*V*_O_]_Raman_), are shown in Table 1. It can be seen that the [*V*_O_]_Raman_ values of the H-Y-, I-Y-, and H/I-Y-doped CeO_2_ were 1.47, 0.93, and 1.16, respectively, which are higher than that of the undoped sample (0.67).

According to the Ce 3d XPS analyses in Figure 4, it can be seen that pure CeO_2_ had a certain number of Ce^3+^ ions, contributing to the OSC of CeO_2_ with the formation and filling of intrinsic *V*_O_ defects, which could be expressed by Equation (3) and written using Kroger and Vink notations as in Equation (4). In the synthesis of CeO_2_, Y ions were introduced and doped into the CeO_2_ lattice, and a substoichiometric CeO_2-*x*_ unit was formed with an increase in *V*_O_ defects. The creation of extrinsic *V*_O_ defects could be expressed by Equations (5) and (6). The vacancy compensation mechanism has been suggested for the increased concentration of *V*_O_ for Y-doping into CeO_2_. As shown in Equations (3) and (4), besides the intrinsic *V*_O_ in CeO_2_, there are two additional kinds of *V*_O_: one *V*_O_ is created to balance the charge when two adjacent Ce^4+^ cations are substituted by two Y^3+^ cations, as shown in Equation (5); and substitution of one Ce^4+^ by one Y^3+^ gives rise to the formation of one *V*_O_ with the adjacent Ce^4+^ reduced to Ce^3+^, as shown in Equation (6).

(3) $$CeO_{2}\overset{Oxygen release}{\underset{Oxygen storage}{⇄}}Ce_{1-x}^{4+}Ce_{x}^{3+}O_{2-x/2}{\left(V_{O}\right)}_{x/2}+x/4O_{2}$$

(4) $${\mathrm{Ce}}_{2}O_{3}\;\overset{{2\mathrm{CeO}}_{2}}{⇄}{\;2\mathrm{Ce}}_{\mathrm{Ce}}^{\prime }+3O_{O}^{\times }+V_{O}^{••}$$

(5) $$Y_{2}O_{3}\;\overset{{2\mathrm{CeO}}_{2}}{⇄}{\;2Y}_{\mathrm{Ce}}^{\prime }+3O_{O}^{\times }+V_{O}^{••}$$

(6) $$Y_{2}O_{3}+{\mathrm{Ce}}_{2}O_{3}\overset{{2\mathrm{CeO}}_{2}}{⇄}{\;Y}_{\mathrm{Ce}}^{\prime }+{\mathrm{Ce}}_{\mathrm{Ce}}^{\prime }+3O_{O}^{\times }+V_{O}^{••}$$

where $Y_{\mathrm{Ce}}^{\prime }$ represents a Y^3+^ cation occupying the site of a Ce^4+^ cation and ${\mathrm{Ce}}_{\mathrm{Ce}}^{\prime }$ represents a Ce^3+^ cation occupying the site of a Ce^4+^ cation. $V_{O}^{}$ and $V_{O}^{••}$ represent an oxygen vacancy defect and one with two positive charges, respectively, which are produced via the vacancy compensation mechanism; and $O_{O}^{\times }$ is a lattice oxygen atom. From the O 1s XPS analysis in Figure 5 and the Raman analysis in Figure 6, it can be seen that pure CeO_2_ crystal itself contained a certain number of *V*_O_ species (namely intrinsic *V*_O_ defects), which exhibited a large deviation from stoichiometry in the atmosphere, forming nonstoichiometric oxide CeO_2−*x*_. After doping with Y^3+^ cations, CeO_2_ could still retain its fluorite crystal structure (see Figure 2), accompanied by the loss of oxygen from its lattice and the consequent formation of a large number of extrinsic *V*_O_ defects (see Table 1, Figure 5 and Figure 6).

Table 2 shows the lattice parameters, [Ce^3+^]_XPS_, [*V*_O_]_XPS_, and [*V*_O_]_Raman_ of undoped and Y-doped CeO_2_ from recent literature [41,42,43]. By comparing these data of undoped and Y-doped CeO_2_, we could find that the lattice expansion occurred upon the incorporation of Y^3+^ into the CeO_2_ lattice, accompanied by the presence of Ce^3+^ ions and more *V*_O_ defects, which were consistent with our results, despite different methods of quantification. However, the relative content for Ce^3+^ ions ([Ce^3+^]_XPS_) decreased with the doping of Y^3+^ in the report [44], and the authors attributed this to the substitution of Y^3+^ for Ce^3+^.

**Figure 7:**
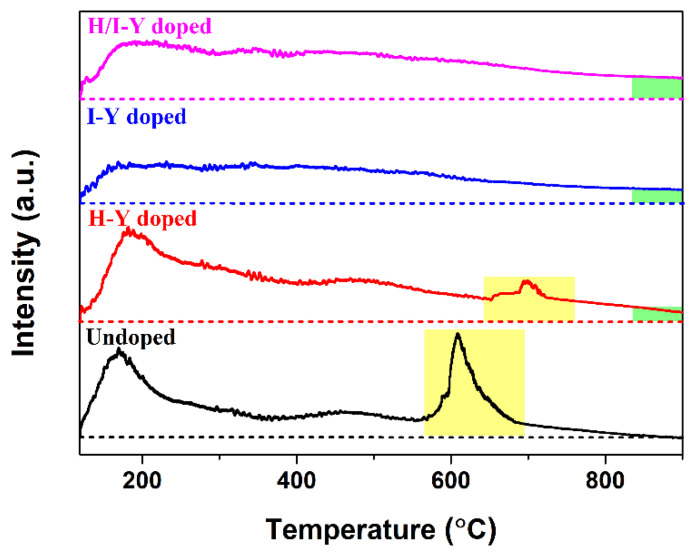
O_2_-TPD profiles of Undoped and H-Y-, I-Y-, and H/I-Y-doped CeO_2_ powders.

OSC is the basic characteristic of CeO_2_ and the premise of numerous applications. Therefore, the O_2_-TPD experiment was employed to evaluate the OSC of CeO_2_. Figure 7 shows the O_2_-TPD spectra of the Undoped and H-Y-, I-Y-, and H/I-Y-doped CeO_2_ powders. For the Undoped CeO_2_, the asymmetrical peak of either the low temperature at ~170 °C or the high temperature at ~600 °C (light yellow area) implied the existence of at least two kinds of oxygen species at various coordination environments. The oxygen desorption at low temperatures could be attributed to the release of surface/subsurface lattice oxygen, while the oxygen desorption at high temperatures could be ascribed to the release of bulk lattice oxygen, which was consistent with the reported results [45]. Moreover, it could be clearly observed that the oxygen desorption was rapid at the early stages of the process (120~170 °C), suggesting that there were large amounts of adsorbed oxygen on CeO_2_, which could emigrate quickly at low temperatures. After the temperature reached ~170 °C, the oxygen desorption started to decrease quickly until a temperature of ~350 °C was reached, and then basically maintained a steady release of oxygen until ~570 °C. Subsequently, the Undoped CeO_2_ experienced the second oxygen release from 570 to 820 °C. Remarkably, the O_2_-TPD curve coincided with the baseline after 820 °C, suggesting that there was little release of oxygen for Undoped CeO_2_ after 820 °C. For the H-Y-doped CeO_2_, the O_2_-TPD profile was similar to that of the Undoped CeO_2_. However, the desorption bands at high temperatures occurred at higher temperatures, indicating that the partial substitution of Ce^4+^ ions (0.97 Å) with the large Y ions (1.02 Å) improved the stability of the CeO_2_ lattice. For the O_2_-TPD profiles of I-Y- and H/I-Y-doped CeO_2_, the asymmetrical peak at high temperatures was displaced by a smooth descent peak, indicating that the oxygen desorption mainly occurred on the surface and subsurface of the I-Y- and H/I-Y-doped CeO_2_ samples. Furthermore, for the O_2_-TPD profiles of the H-Y-, I-Y-, and H/I-Y-doped CeO_2_ after 820 °C, there were still some distances to the baseline (light green area), indicating sustaining oxygen desorption, which could be attributed to the formation of extrinsic *V*_O_ defects in the interior of the CeO_2_ lattice caused by Y-doping.

For comparison purposes, the OSC was quantified by the amount of O_2_ desorption per gram of CeO_2_ sample by measuring the corresponding peak areas of O_2_-TPD profiles. The quantified OSC values (labeled as [OSC]) of the Undoped and H-Y-, I-Y-, and H/I-Y-doped CeO_2_ in the full temperature range are shown in Table 1. Compared with the OSC value of the Undoped CeO_2_, all of the Y-doped CeO_2_ samples were enhanced, and the sequence of [OSC] was as follows: H-Y-doped CeO_2_ (0.372 mmol/g) > H/I-Y-doped CeO_2_ (0.353 mmol/g) > I-Y-doped CeO_2_ (0.248 mmol/g) > Undoped CeO_2_ (0.153 mmol/g). The OSC of the H-Y-, I-Y-, and H/I-Y-doped CeO_2_ were increased by 143.1%, 62.1%, and 130.7%, respectively, compared with that of the Undoped CeO_2_. The enhanced OSC of all the Y-doped CeO_2_ could be explained as follows: when Y^3+^ ions were doped into the CeO_2_ lattice to substitute Ce^4+^ ions, the extrinsic *V*_O_ defects were formed to keep the electric neutrality of their fluorite structure, accompanied by the increase in the number of oxidation/reduction cycles composed of Ce^3+^ and Ce^4+^ states (Ce^3+^↔Ce^4+^). Y-doping of CeO_2_ possesses both intrinsic and extrinsic *V*_O_ defects, as well as the oxidation/reduction cycle of Ce^3+^↔Ce^4+^, which could determine the transfer of oxygen ions and OSC.

## 4. Conclusions

In summary, three various routes were adopted to successfully synthesize Y-doped CeO_2_ solid solutions. The large Y cations were incorporated into the CeO_2_ lattice with normal trivalence and formed a Y-doped CeO_2_ solid solution while holding the cubic fluorite structure of CeO_2_. The results of O 1s XPS and Raman spectroscopy indicated that pure CeO_2_ crystal itself contained a certain number of intrinsic *V*_O_ defects. With the substitution of Ce^4+^ ions with Y^3+^ ions in the CeO_2_ lattice, local lattice expansion of CeO_2_ crystal occurred, extrinsic *V*_O_ defects were formed, and there was an increase in the number of oxidation/reduction cycles composed of Ce^3+^ and Ce^4+^ states. Moreover, the relative concentrations of *V*_O_ defects were quantified by the *A*_592_/*A*_464_ values obtained from Raman spectra, which were 1.47, 0.93, and 1.16 for the H-Y-, I-Y-, and H/I-Y-doped CeO_2_, respectively, and were higher than that of the undoped one (0.67). There were large amounts of adsorbed oxygen on CeO_2_, which could emigrate quickly at low temperatures, and the OSCs of the H-Y-, I-Y-, and H/I-Y-doped CeO_2_ were increased by 143.1%, 62.1%, and 130.7%, respectively, compared with that of the Undoped CeO_2_ (0.153 mmol O_2_/g CeO_2_). Both the intrinsic and extrinsic *V*_O_ defects, as well as the oxidation/reduction cycle of Ce^3+^↔Ce^4+^, could determine the enhanced OSC of Y-doped CeO_2_. The CeO_2_ with doping during only the hydrothermal process exhibited the maximum values of OSC, suggesting the effectiveness of the doping.

## Figures and Tables

**Table 1:** Phase, lattice parameters, [Ce^3+^]_XPS_, [*V*_O_]_XPS_, [*V*_O_]_Raman_, and [OSC] of Undoped and H-Y-, I-Y-, and H/I-Y-doped CeO_2_.

Parameter Sample Name	Undoped	H-Y Doped	I-Y Doped	H/I-Y Doped
Phase from XRD	CeO_2_	CeO_2_	CeO_2_	CeO_2_
Lattice parameters (Å)	5.4117	5.4242	5.4190	5.4227
[Ce^3+^]_XPS_ (%)	6.54	12.60	8.95	11.37
[*V*_O_]_XPS_ (%)	24.36	30.65	26.32	28.72
[*V*_O_]_Raman_	0.67	1.47	0.93	1.16
[OSC] (mmol O_2_/g CeO_2_)	0.153	0.372	0.248	0.353

**Table 2:** Recent literature on the lattice parameters, [Ce^3+^]_XPS_, [*V*_O_]_XPS_, and [*V*_O_]_Raman_ of undoped and Y-doped CeO_2_.

Authors	Lattice Parameters (Å)	[Ce^3+^]_XPS_ (%)	[*V*_O_]_XPS_ (%)	[*V*_O_]_Raman_
Xu et al. [41]	5.4178 (CeO_2_); 5.4217 (Y-doped)	12.60 (CeO_2_); 21.38 (Y-doped)	28.53 (CeO_2_); 33.81 (Y-doped)	1.1 (CeO_2_); 4.9 (Y-doped)
Chahal et al. [42]	5.413 (CeO_2-δ_); 5.416 (Ce_0.97_Y_0.03_O_2-δ_)	26.6% (CeO_2-δ_); 35.3% (Ce_0.97_Y_0.03_O_2-δ_)	46.9% (CeO_2-δ_); 52.6% (Ce_0.97_Y_0.03_O_2-δ_)	1.9 (CeO_2-δ_); 2.9 (Ce_0.97_Y_0.03_O_2-δ_)
Yang et al. [43]	5.4129 (CeO_2_); 5.4325 (Y-doped)	18.13% (CeO_2_); 22.88% (Y-doped)	/	/
Liyanage et al. [44]	/	11.40 (CeO_2_); 10.07 (Ce_0.89_Y_0.11_O_1.94_)	/	/

## Data Availability

Not applicable.
